# Association of clinical variables and thyroid-stimulating hormone with psychotic symptoms in patients with first-episode and drug-naïve major depressive disorder with elevated fasting blood glucose: preliminary exploratory study with a large sample

**DOI:** 10.1192/bjo.2024.53

**Published:** 2024-05-03

**Authors:** Qian Yang, Qianjin Wang, Pu Peng, Tieqiao Liu, Xiangyang Zhang

**Affiliations:** Department of Psychiatry, National Clinical Research Center for Mental Disorders, and National Center for Mental Disorders, The Second Xiangya Hospital of Central South University, Changsha, China; and Department of Psychology, Zhongshan City People's Hospital, Zhongshan, China; Department of Psychiatry, National Clinical Research Center for Mental Disorders, and National Center for Mental Disorders, The Second Xiangya Hospital of Central South University, Changsha, China; CAS Key Laboratory of Mental Health, Institute of Psychology, Chinese Academy of Sciences, Beijing, China

**Keywords:** Depressive disorder, first-episode and drug-naïve, psychotic symptoms, thyroid-stimulating hormone, elevated fasting blood glucose

## Abstract

**Background:**

Psychotic symptoms and elevated fasting blood glucose (FBG) are frequently observed in people with major depressive disorder (MDD), but there is a lack of research into this relationship within this cohort.

**Aims:**

This study aimed to preliminarily explore the prevalence of psychotic symptoms and their predictors among patients with MDD and elevated FBG.

**Method:**

This study enrolled 1718 patients with first-episode and drug-naïve (FEDN) MDD. Sociodemographic data and physical and biochemical indicators were collected. Clinical symptoms were assessed with tools such as the Hamilton Rating Scale for Anxiety, Hamilton Rating Scale for Depression (HRSD) and Positive and Negative Syndrome Scale positive subscale.

**Results:**

The odds ratio for psychotic symptoms in those with MDD and elevated FBG (18.7%) was 2.33 times higher than those with MDD without elevated FBG. Presence of psychotic symptoms was significantly correlated with HRSD score, suicide attempts, and total cholesterol and thyroid-stimulating hormone levels. The combination of HRSD score, suicide attempts and thyroid-stimulating hormone levels among patients with MDD and elevated FBG effectively distinguished between individuals with and without psychotic symptoms, achieving an area under the curve of 0.87.

**Conclusions:**

Psychotic symptoms are frequently observed among FEDN MDD patients with elevated FBG, and depressive symptoms, suicide attempts and thyroid-stimulating hormone levels are related to psychotic symptoms in this cohort.

Major depressive disorder (MDD) ranks among the most prevalent mental illnesses globally, affecting 3.6% of Chinese adults within a 1-year period and 6.9% over their lifetime.^1^ The Global Burden of Disease Study 2019 highlights mental disorders as persisting within the top ten causes of the global disease burden, with MDD constituting the highest proportion.^2^ Findings from a longitudinal study reveal that individuals with MDD exhibit a 3.2-fold increase in the utilisation of out-patient healthcare resources, an 8.6-fold rise in work loss and a one-fold elevation in all-cause mortality compared with matched groups.^3^

## The bidirectional link between MDD and elevated fasting blood glucose (FBG)

In recent years, mounting evidence has indicated a bidirectional relationship between MDD and elevated FBG, consequently amplifying the global medical burden and patient mortality.^4–8^ For instance, Mezuk et al conducted a prospective meta-analysis revealing that individuals with MDD face a 60% higher lifetime risk of developing type 2 diabetes.^4^ Similarly, Koponen et al identified a notable association between suicidal behaviour and impaired glucose metabolism in patients with MDD.^8^ These outcomes, however, could be influenced by the disease's progression and the treatment administered. Concentrating on individuals with first-episode and drug-naïve (FEDN) MDD aids in reducing biases associated with potential confounding factors such as disease duration and medication use.^9^

## Psychotic symptoms in MDD: prevalence and impact

Psychotic symptoms are highly prevalent among patients with MDD, but the prevalence varies by population, diagnostic criteria and regions. Extensive research conducted in psychiatric wards of 13 hospitals across China showed a 9.2% rate of psychotic symptoms in MDD cases.^10^ In stark contrast, the French community reports indicated that up to 39.3% of patients with MDD experienced at least one psychotic symptom (either hallucinations or delusions).^11^ Additionally, our latest study revealed that psychotic symptoms occurred in 10% of the FEDN MDD population.^12^ It is noted that patients with MDD exhibiting psychotic symptoms suffer from more intense depressive symptoms, frequent depressive episodes, heightened social impairment, increased co-occurrence with other mental disorders and a higher suicide rate.^10,11,13^ Previous findings also highlight the significant difference in treatment strategies for patients with MDD with and without psychotic symptoms, emphasising the importance of distinguishing between psychotic and non-psychotic forms of depression for effective therapy.^14^ Despite these insights, the psychopathological mechanisms behind these symptoms are yet to be fully understood.

It is widely acknowledged that psychotic symptoms is prevalent not only in individuals with psychiatric disorders,^15^ but also in those with abnormal glucose metabolism.^16^ Limited research has explored psychotic symptoms among individuals with psychiatric conditions who also have elevated FBG, with a predominant focus on schizophrenia and bipolar disorder.^17,18^ However, investigations into clinical symptoms and their predictors among patients with MDD with elevated FBG have predominantly centred on suicide attempts^19,20^ rather than psychotic symptoms. Consequently, it is imperative to scrutinise the occurrence of psychotic symptoms and their adverse outcomes among patients with MDD with elevated FBG in this study. Drawing from previously established evidence, this study aims to preliminarily investigate the prevalence and clinical relevance of psychotic symptoms in individuals with comorbid fasting elevated blood glucose and MDD, employing an analysis of thyroid hormone levels and metabolic markers.

## Method

### Patients

From 2015 to 2017, this study recruited 1718 individuals diagnosed with FEDN MDD at the psychiatric out-patient clinic of the First Clinical Medical College of Shanxi Medical University. Participants in this study had to meet specific inclusion and exclusion criteria. Inclusion criteria comprised (a) a diagnosis of MDD according to the DSM-IV; (b) 17-item Hamilton Rating Scale for Depression (HRSD) score ≥24; (c) aged between 18 and 60 years; (d) acute first episode of MDD, with no prior use of psychotic or specialised medication; and (e) Han nationality. Exclusion criteria included (a) pregnant or breastfeeding women; (b) patients with significant physical illnesses (e.g. organic brain diseases, immune system illnesses); (c) individuals with documented substance misuse (except for smoking), based on self-report or medical records; and (d) refusal to provide informed consent.

### Demographic characteristics

To gather sociodemographic information, participants completed self-report questionnaires detailing their age, gender, height, weight, marital status and level of education.

### Clinical interview and assessment

The assessment of anxiety and depressive symptoms, along with the presence of psychotic symptoms, was conducted with the Hamilton Rating Scale for Anxiety (HRSA), HRSD and the Positive and Negative Syndrome Scale (PANSS) positive subscale, respectively. Criteria for identifying anxiety symptoms, depressive symptoms and psychotic symptoms involved scores of 18 or more on the HRSA, 24 or more on the HRSD and 15 or above on the PANSS positive subscale.^21–23^ Structured clinical evaluations were carried out by two expertly trained psychiatrists, achieving interrater reliability coefficients exceeding 0.8 across the HRSD, HRSA and PANSS positive subscale.

In terms of evaluating suicide attempts, any action taken by study participants with a intention to end their own lives was categorised as such.^24^ The assessment of suicide attempts was based on interviews, beginning with the question, ‘Have you ever attempted suicide?’. A positive response was recorded as a suicide attempt, and additional information was subsequently collected regarding the method, frequency and dates of these attempts. In cases where responses were ambiguous, further verification was sought from acquaintances, such as family members or friends, to ensure the accuracy of the reported incidents.

### Physical and biochemical parameter measurements

On the evening before their evaluation, all participants observed a fasting period and then underwent serum sampling between 06.00h and 08.00h the following day. The serum samples were analysed for various fasting biochemical indicators, including free triiodothyronine (FT3), free thyroxine (FT4), thyroid peroxidase antibodies (TPOAb), anti-thyroglobulin antibodies (TGAb), thyroid-stimulating hormone (TSH), total cholesterol, triglycerides, high-density lipoprotein cholesterol (HDL-C), low-density lipoprotein cholesterol (LDL-C) and FBG. All serum samples were tested before 11.00h. FBG levels were considered elevated if they reached or exceeded 6.1 mmol/L.^25^ The body mass index calculation was performed by dividing the weight of the patient in kilograms by their height in meters squared. Blood pressure, both systolic and diastolic, was measured by a certified nurse using a sphygmomanometer, with the patient in a lying down position.

### Statistical analysis

Statistical evaluations were conducted with SPSS version 26.0 for Windows, adopting a two-tailed significance level of 0.05, and graphical representations were created with GraphPad Prism version 8.0 for Windows (GraphPad Software, San Diego, CA; see https://www.graphpad.com/). To assess the normality of the data-set, the Kolmogorov–Smirnov test was employed. The analysis of categorical and continuous data was carried out with the chi-squared test and either the analysis of variance or the Mann–Whitney *U*-test, as appropriate. A univariate analysis aimed to identify potential predictors for psychotic symptoms among patients with FEDN MDD with elevated FBG. Variables that showed a significant association with psychotic symptoms in the univariate analysis (*P* < 0.05) were further examined in a multivariable logistic regression analysis using the backward Wald method. The capability of the significant factors to distinguish between patients with and without psychotic symptoms was assessed through receiver operating characteristic curves, with a threshold above 0.7 considered satisfactory. Given the study's exploratory phase, adjustments for multiple testing were not applied. The presence of multicollinearity among the independent variables was evaluated with the variance inflation factor (VIF), with a VIF > 5 indicating significant multicollinearity.

### Ethics statement

The authors declare that all practices related to this study adhere to the ethical guidelines of the pertinent national and institutional bodies governing human experimentation, aligning with the Helsinki Declaration of 1975, as amended in 2008. The Institutional Review Board of the First Clinical Medical College of Shanxi Medical University granted approval for all procedures relating to human participants (approval number 2016-Y27). All participants were fully informed about the study's objectives and methods, and provided their written consent.

## Results

### Prevalence of psychotic symptoms among patients with MDD with and without elevated FBG

The prevalence of elevated FBG in patients with MDD was 14% (241/1718). Patients with elevated FBG exhibited significantly higher scores in the PANSS positive subscale, HRSD and HRSA compared with those without elevated FBG (all *P* < 0.001). Moreover, among patients with MDD with elevated FBG, the prevalence of psychotic symptoms was 18.7% (45/241), which was significantly higher than in those without elevated FBG (8.5%, 126/1477) (*χ*^2^ = 23.78, *P* < 0.001; odds ratio 2.46, 95% CI 1.70–3.57). After adjusting for sociodemographic variables, including age, age at onset, gender, duration of illness, marital status and education level, the odds ratio for psychotic symptoms among patients with MDD with elevated FBG remained significantly higher, at 2.33 (β = 0.85, *P* < 0.001; odds ratio 2.33, 95% CI 1.60–3.40).

### Clinical traits and biochemical markers in patients with MDD with elevated FBG, with and without psychotic symptoms

Table 1 illustrates that patients with MDD with psychotic symptoms exhibited elevated scores in the HRSD, HRSA and PANSS positive subscale, along with a higher prevalence of anxiety symptoms and an increased rate of suicide attempts (all *P* < 0.05), compared with patients with MDD without psychotic symptoms. Furthermore, systolic and diastolic blood pressure, total cholesterol and TSH levels were significantly higher in patients with MDD with psychotic symptoms than in those without psychotic symptoms (all *P* < 0.05). Given the initial investigative nature of this research, the findings presented in Table 1 were not adjusted for multiple statistical comparisons.

**Table 1:** Sociodemographic and clinical characteristics between major depressive disorder comorbid elevated fasting blood glucose with and without psychotic symptoms

	MDD comorbid elevated fasting blood glucose			
	Without psychotic symptoms (*n* = 196)	With psychotic symptoms (*n* = 45)	Statistic value	*P-*value
Age, years	32.0 ± 22.0	42.0 ± 25.0	*z* = −1.23	0.22
Gender			*x*^2^ = 0.18	0.67
Female	134 (68.4%)	32 (71.1%)		
Male	62 (31.6%)	13 (28.9%)		
Education			*x*^2^ = 4.88	0.18
Middle school	52 (26.5%)	18 (40.0%)		
High school	89 (45.4%)	15 (33.3%)		
College	45 (23.0%)	8 (17.8%)		
Graduate	10 (5.1%)	4 (8.9%)		
Marital status			*x*^2^ = 0.01	0.91
Single	54 (27.6%)	12 (26.7%)		
Married	142 (72.4%)	33 (73.3%)		
Age at onset, years	32.0 ± 21.3	41.0 ± 25.0	*z* = −1.20	0.23
Illness duration, months	5.00 ± 5.00	6.00 ± 3.00	*z* = −1.78	0.08
HRSD	31.0 ± 4.00	33.0 ± 3.00	*z* = −5.74	<0.001
HRSA	21.0 ± 4.00	27.0 ± 4.00	*z* = −8.81	<0.001
Psychosis positive score	7.00 ± 1.00	21.0 ± 3.00	*z* = −11.83	<0.001
Severe anxiety symptoms	172 (87.8%)	45 (100.0%)	*F* = 6.12	0.01
Suicide attempts			*F* = 28.57	<0.001
No	140 (71.4%)	13 (28.9%)		
Yes	56 (28.6%)	32 (71.1%)		
BMI, kg/m^2^	24.4 ± 2.58	24.5 ± 2.32	*z* = −0.06	0.96
Systolic blood pressure, mmHg	123 ± 11.5	126 ± 17.0	*z* = −2.74	0.006
Diastolic blood pressure, mmHg	78.0 ± 9.00	80.0 ± 11.0	*z* = −2.37	0.018
Total cholesterol, mmol/L	5.67 ± 1.61	6.21 ± 1.48	*F* = 5.82	0.02
Triglycerides, mmol/L	2.27 ± 1.33	2.62 ± 1.39	*z* = −1.37	0.17
HDL-C, mmol/L	1.15 ± 0.49	1.02 ± 0.36	*F* = 1.32	0.19
LDL-C, mmol/L	3.26 ± 1.24	3.21 ± 0.86	*z* = −0.48	0.63
TSH, mmol/L	6.87 ± 3.65	9.36 ± 1.90	*z* = −6.47	<0.001
TgAb, IU/L	23.1 ± 79.1	23.2 ± 158	*z* = −1.35	0.18
TPOAb, IU/L	19.6 ± 73.3	26.2 ± 209	*z* = −1.36	0.17
FT4, mmol/L	17.0 ± 3.09	16.5 ± 2.49	*F* = 1.01	0.32
FT3, mmol/L	4.90 ± 0.925	4.86 ± 1.12	*z* = −0.41	0.68

MDD, major depressive disorder; HRSD, Hamilton Rating Scale for Depression; HRSA, Hamilton Rating Scale for Anxiety; BMI, body mass index; HDL-C, high-density lipoprotein cholesterol; LDL-C, low-density lipoprotein cholesterol; TSH, thyroid-stimulating hormone; TgAb, anti-thyroglobulin antibody; TPOAb, thyroid peroxidases antibody; FT4, free thyroxine; FT3, free triiodothyronine.

### Predictors for psychotic symptoms among patients with MDD with elevated FBG

Next, we explored potential predictors for psychotic symptoms among patients with MDD with elevated FBG. The search for predictors of psychotic symptoms within this group, characterised by disrupted glucose regulation, entailed logistic regression analysis (backward Wald method) focusing on variables distinctly impactful in multivariable analyses.

**Fig. 1:**
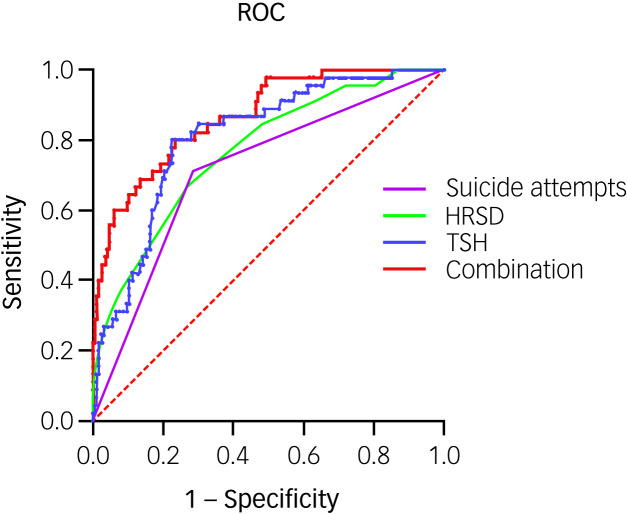
The discriminatory capacity of related factors for distinguishing between patients with and without psychotic symptoms in major depressive disorder comorbid with elevated fasting blood glucose. The area under the curve of suicide attempts, HRSD score, TSH levels and the combination of these three factors was 0.71, 0.77, 0.81 and 0.87, respectively. HRSD, Hamilton Rating Scale for Depression; ROC, receiver operating characteristic; TSH, thyroid-stimulating hormone.

Table 2 reveals key factors linked to psychotic symptoms in patients with MDD with elevated FBG, including HRSD score (*P* = 0.001; odds ratio 1.55, 95% CI 1.27–1.90), suicide attempts (*P* = 0.011; odds ratio 3.01, 95% CI 1.29–7.02), total cholesterol (*P* = 0.013; odds ratio 0.56, 95% CI 0.35–0.89) and TSH (*P* = 0.001; odds ratio 1.69, 95% CI 1.35–2.11) levels. This investigation highlighted a VIF <5 for all variables, suggesting no multicollinearity concerns. Additionally, the area under the curve (AUC) for these predictors was noted as follows: 0.77 for HRSD score, 0.71 for suicide attempts, 0.61 for total cholesterol and 0.81 for TSH; AUC values >0.7 were deemed satisfactory. By integrating data on HRSD score, suicide attempts and TSH levels, we discerned a significant discriminative power for identifying psychotic symptoms presence in patients with MDD with elevated FBG, evidenced by an AUC of 0.87 (*P* < 0.001; 95% CI 0.81–0.92), as shown in Fig. 1. In addition, to fully demonstrate the scientific validity and accuracy of the study, the Supplementary Materials available at https://doi.org/10.1192/bjo.2024.53 show the comparative results regarding psychotic symptoms-related factors in patients with MDD with elevated fasting glucose without Bonferroni correction. Details are shown in Supplementary Tables 1 and 2.

**Table 2:** Factors associated with psychotic symptoms in patients with major depressive disorder and elevated fasting blood glucose

	*P*-value	Odds ratio	95% CI
HRSD score	<0.001	1.55	1.27–1.90
Suicide attempts	0.011	3.01	1.29–7.02
TSH	<0.001	1.69	1.35–2.11
Total cholesterol	0.013	0.56	0.35–0.89

HRSD, Hamilton Rating Scale for Depression; TSH, thyroid-stimulating hormone.

## Discussion

To the best of our knowledge, this study represents the inaugural comprehensive analysis assessing the prevalence and determinants of psychotic symptoms in Chinese Han patients with FEDN MDD and elevated FBG. Our main findings were as follows: (a) the odds ratio of psychotic symptoms was 2.33 times greater among patients with FEDN MDD and elevated FBG than those without elevated FBG; (b) psychotic symptoms were significantly correlated with HRSD score, suicide attempts, total cholesterol and TSH levels; and (c) combining HRSD score, suicide attempts and TSH levels of patients with FEDN MDD with elevated FBG could effectively distinguish patients with and without psychotic symptoms (AUC of 0.87).

So far, several research efforts have identified a connection between elevated FBG and the occurrence of psychotic symptoms, but none have found the prevalence of psychotic symptoms among patients with MDD with elevated FBG.^26–29^ For instance, research conducted in Australia involving a substantial cohort of 1642 psychiatric patients revealed a significant correlation between a familial history of diabetes and a similar background in psychiatric conditions among individuals with mental health disorders.^29^ More recently, Chouinard et al also found that abnormalities in glucose metabolism and insulin signalling were associated with risk and manifestations of psychiatric disorders, not only attributable to the treatment of the disorder or the lifestyle of the patients.^27^ Another study among patients with bipolar disorder found that people with diabetes tended to use antipsychotics more frequently than those without diabetes (72% *v.* 57%), although this difference was not significantly different.^28^ Previously, Ceretta et al found that after adjustment for other variables (e.g. age, gender, etc.), the risk of psychotic mood disorders in patients with diabetes and those with higher glycated haemoglobin (after fasting glucose control) was 2.5 times and 4.1 times higher, respectively.^26^ Our current findings were in agreement with the majority of earlier investigations, suggesting that elevated FBG was strongly and statistically significantly associated with the presence of psychotic symptoms. However, Habtewold et al discovered no link between elevated glycated haemoglobin and the development of psychiatric disorders.^30^ Our current understanding suggests that the various results indicated above could be the consequence of the following factors. First, sample heterogeneity in studies, such as the disease's phases (acute or stable)^31^ and the count of depressive episodes,^32^ may have influenced the results. Furthermore, a European multicentre investigation conducted by Dold et al noted that patients with MDD exhibiting melancholic features showed more pronounced psychotic symptoms than those without such features.^33^ Second, different choices of treatment for patients with MDD have different effects on treatment, such as whether to choose a combination of antipsychotic medications.^34,35^ Third, certain antipsychotics (such as clozapine, olanzapine, risperidone, etc.) have the side-effect of increasing blood glucose, which may exaggerate the association with blood glucose metabolism.^36^ Consequently, our research focused on recruiting patients with FEDN MDD with FBG, aiming to reduce the impact of confounders like the number of disease episodes and medication effects on the results.

The current investigation discovered that various clinical and biochemical markers, including the HRSD score, suicidal thoughts, TSH levels and total cholesterol levels, were linked to psychotic symptoms among patients with FEDN MDD and elevated FBG. Previous studies have consistently identified suicide attempts and depressive symptoms in patients with MDD as important predictors for psychotic symptoms. For instance, some of our previous studies showed that psychotic symptoms in FEDN MDD was significantly associated with HRSD score and suicide attempts.^12,21^ Gaudiano et al^37^ discovered that patients with psychotic major depression experienced increased severity of depressive symptoms, higher frequency of suicidal ideation and greater impairments in social and occupational functioning. Moreover, similar findings have been found in other populations. For example, depressive symptoms were found to be associated with psychotic symptoms in individuals with a dual diagnosis of psychiatric disorders and substance misuse.^38^ Prochwicz et al^39^ also determined that depression and anxiety served as mediators in the relationship between temperament, personality and psychiatric-like experiences in healthy individuals. Furthermore, TSH and total cholesterol levels were identified as significant predictors for psychotic symptoms in the present study. In patients with FEDN MDD, our prior research demonstrated a link between PANSS positive subscale score and TSH levels.^40^ However, Contreras et al^41^ found no difference in TSH levels between psychiatric and non-psychiatric patient groups, because of the heterogeneity of the clinical sample. As a result, future research should focus on the function of TSH levels in psychotic symptoms among patients with MDD.

To our understanding, a solitary investigation has explored the association between lipid metabolism disturbances and psychotic symptoms among patients with FEDN MDD. Specifically, Wang et al^42^ identified triglyceride concentrations in individuals with FEDN MDD as an independent predictor of psychotic symptoms. Moreover, a longitudinal study over 5 years demonstrated that patients with higher initial triglyceride levels experienced intensified psychotic symptoms.^43^ Gohar et al^44^ also established a positive correlation between elevated total cholesterol and LDL-C levels and depression among patients with psychosis. The evidence presented above strongly suggests that dyslipidaemia and greater psychotic symptom severity are related. Furthermore, extensive research has revealed a significant relationship between heightened total cholesterol levels and insulin resistance in individuals experiencing first-episode schizophrenia,^45,46^ indicating that lipid metabolism irregularities may also influence insulin resistance or glucose metabolism, particularly in individuals with psychiatric conditions linked to psychotic symptoms. Additionally, an investigation found that individuals with schizophrenia or mood disorders, whether bipolar or unipolar, exhibited elevated total cholesterol, LDL-C, triglyceride and hyperglycaemia levels, and diminished HDL-C levels,^47^ highlighting that dyslipidaemia extends beyond patients with MDD. Thus, future research should not only concentrate on severe psychotic symptoms in mental health conditions, but also systematically evaluate lipid profiles.

This investigation faces several limitations. First, this is a cross-sectional study, and we have only preliminary insights into the prevalence and clinical relevance for psychotic symptoms among patients with FEDN MDD with elevated FBG. Future longitudinal studies are essential to establish causal links between clinical variables and psychotic symptoms in patients with MDD with disturbed glucose metabolism, highlighting the importance of symptom onset order. Second, this investigation exclusively enrolled Chinese Han out-patients diagnosed with MDD without comorbidities. Consequently, for broader generalisability, it is imperative to validate our findings in populations characterised by diverse racial and clinical backgrounds. Third, the participants of this study were adult patients aged 18–60 years; however, age is an important factor affecting blood glucose, which may lead to some biases in our findings. In addition, because of the exploratory nature of this study, no correction for multiple testing was performed, so our results need to be confirmed in a larger population.

In conclusion, the current investigation suggests that patients with FEDN MDD with elevated FBG are associated with a higher likelihood of having psychotic symptoms compared with patients without elevated FBG. Depressive symptoms, suicide attempts, TSH levels and total cholesterol levels are all strong predictors for psychotic symptoms in patients with FEDN MDD and elevated FBG. Investigating the frequency of psychotic symptoms and its determinants in this subgroup can shed light on its pathogenesis, and provide data for clinical interventions and research in this population group.

## Supporting information

Yang et al. supplementary material Yang et al. supplementary material

## Data Availability

The data that support the findings of this study are available from the corresponding author, T.L., upon reasonable request.
