# Supplementary material for: Association of clinical variables and thyroid-stimulating hormone with psychotic symptoms in patients with first-episode and drug-naïve major depressive disorder with elevated fasting blood glucose: preliminary exploratory study with a large sample
Source: BJPsych Open. 2024 May 3;10(3):e99. doi: 10.1192/bjo.2024.53 (PMC11094438; doi:10.1192/bjo.2024.53)
Supplement: Yang et al. supplementary material [file S205647242400053Xsup001.docx]

Supplementary Tables 1 and 2 show the comparative results of factors associated with psychiatric symptoms in MDD patients with elevated FBG, without and with Bonferroni correction, respectively.

Supplementary Table 1 shows the results without Bonferroni correction (p < 0.05) for factors associated with psychiatric symptoms in MDD patients with elevated FBG.

|  | *P* | OR | 95%CI |
| --- | --- | --- | --- |
| HAMD score | <0.001 | 1.55 | 1.27-1.90 |
| Suicide attempts | 0.011 | 3.00 | 1.29-7.00 |
| TSH | <0.001 | 1.70 | 1.33-2.15 |
| TC | 0.013 | 0.56 | 0.35-0.88 |
| SBP | 0.69 | 1.01 | 0.95-1.08 |
| DBP | 0.65 | 0.98 | 0.90-1.07 |

*Note*: HAMD = Hamilton Depression Rating Scale, TSH = thyroid-stimulating hormone, TC = total cholesterol.

Supplementary Table 2 shows the Bonferroni-corrected results (p < 0.05/21 = 0.00238) for factors associated with psychiatric symptoms in MDD patients with elevated FBG.

|  | *P* | OR | 95%CI |
| --- | --- | --- | --- |
| HAMD score | <0.001 | 1.43 | 1.19-1.72 |
| Suicide attempts | 0.016 | 2.76 | 1.21-6.28 |
| TSH | <0.001 | 1.57 | 1.27-1.95 |

*Note*: HAMD = Hamilton Depression Rating Scale, TSH = thyroid-stimulating hormone, TC = total cholesterol.

It should be noted that when performing binary logistic regression, we found that the ORs for the dichotomous variable (i.e., the cut-off point for determining the presence of anxiety symptoms in terms of whether or not the HAMA was greater than 18 points) were significantly abnormal (very large).

On the one hand, this suggests that there is really a strong association between anxiety and the predicted response variable, and its OR may become very large, indicating that the variable has a strong influence on the probability of the predicted response variable. However, on the other hand, when the OR is very large, it needs to be interpreted with caution as it may suggest the possibility of other problems. Also, issues such as outliers, multicollinearity, and sample imbalance in the data may affect the model results.

Based on the above issues, we fully examined the data and did not find any outliers in the data. Meanwhile, we tested the multicollinearity between the variables and found that the VIF were less than 5, which indicated the lack of covariance between the variables. Conversely, when testing for sample imbalance, it was found that the number of MDD patients with anxiety who developed psychotic symptoms (PS) was 45 and the number of those who did not develop PS was 0, as detailed in Table 1 in the main text. Sample imbalance may be the main reason for the large OR for the anxiety variable, which would require studies with larger sample sizes to validate and eliminate this result. Therefore, we did not include the dichotomous variable of presence of anxiety in the supplementary analyses of the subsequent binary logistic regression.

This decision was made primarily because the model also actively excluded the binary variable of whether or not there was anxiety from the binary logistic regression when we used the Backward (Wald) method, which did not affect the other results, as detailed in the results in Table 2 in the main text.
